# INTRAPEC Technique Controls Pectoralis Spasm and Pain for Subpectoral Breast Implantation: A Retrospective Study

**DOI:** 10.1097/GOX.0000000000002646

**Published:** 2020-02-26

**Authors:** Jonathan Kline, Wayne Lee, Ken Wofford

**Affiliations:** From * Twin Oaks Anesthesia, LLC, Wesley Chapel, Fla.; † Wayne Lee Plastic Surgery Center; ‡ University of South Florida, Tampa, Fla.

## Abstract

**Background:**

In 2018, a novel approach to reduce pectoralis spasm from sub- pectoral breast implant surgery was published called the INTRAPEC.1 In this study, we more formally examine the effectiveness of the ultrasound-guided INTRAPEC injection to control postoperative pectoralis major spasm and pain following breast surgery with sub-pectoral implantation.

**Methods:**

We employed a simple postoperative spasm and pain record to collect data on 17 patients, all of whom received INTRAPEC and erector spinae plane blocks as a part of an opioid- free anesthetic. All breast surgeries were completed with LMA general anesthesia, preserving spontaneous ventilation.

**Results:**

Of the 17 study participants, 13 (76.4%) reported spasm scores less than 3 for the entire 2-day study period and, at most time points, patients reported a median score for pain of 2, with IQRs ranging from 1 to 7.

**Conclusions:**

The study results suggest that the INTRAPEC injection is a simple, low-cost, low-risk, and effective technique that controls post- operative spasm following breast surgery involving sub-pectoral implantation.

## INTRODUCTION

Approximately 50% of patients develop chronic pain after breast surgery.^2,3^ Several case reports highlight the role of postoperative pectoralis muscle spasm in the development of chronic pain after breast surgery.^2^ Subpectoral implantation can be part of major breast reconstruction following mastectomy for cancer surgery, particularly phase 2, as well as straightforward cosmetic breast augmentation.

Previous efforts to ameliorate pectoralis spasm-related postoperative pain have involved intraoperative botulinum toxin injection, postoperative neurectomy of the medial and lateral pectoral nerve, and/or pre- or postoperative blockade of the medial and lateral pectoral nerve.^5^ The risk of postoperative pectoralis spasm contributing to the development of chronic pain makes prophylactic control of spasm and pain after subpectoral implantation a priority for anesthesia providers. Given evidence-driven and/or state-mandated initiatives to decrease or avoid perioperative opioid use, regional blockade of the medial and lateral pectoral nerve has numerous advantages for perioperative pain management of the patient undergoing subpectoral implantation. The INTRAPEC technique, introduced in 2018, can be used for a variety of breast surgeries such as revision of existing implants or first-time breast augmentation spasm control.^1^ The advantages over traditional techniques, such as the PECs 1 block, make this technique appealing. Other techniques exist to treat postoperative spasms but have not been formally studied in their effectiveness in spasm prevention. The introduction of the PECs 1 technique by R. Blanco was a significant step forward in targeted pectoralis muscle pain as it sought to block the muscle spasm at the nerve level. The technique involves a discrete placement of local anesthetic between the pectoralis major and minor, to hopefully contact and block the median and lateral pectoral nerves.^4^ However, due to the lack of consistency in anatomical placement of these nerves, even an ultrasound-guided injection correctly placed between the muscle planes will not guarantee a nerve block. The presence of existing subpectoral implants also makes this technique challenging and somewhat risky, even in experienced hands. A subtle needle advancement causing intrusion into an implant could have considerable consequences. Importantly, regarding the placement of the PECs 1 technique, the surgically induced implant pocket comprises the exact location of the local anesthetic deposition. As a consequence of this, before final implantation, a washout of this pocket by the surgical team means a dramatic reduction of any local anesthetic deliberately placed there. This inevitably will cause a significant reduction in blockade of the median and lateral pectoral nerves. Lastly, unlike the INTRAPEC, the PECs 1 block fails to provide intraoperative muscle compliance during electrocautery. These reasons make the PECs 1 technique virtually obsolete in its application for subpectoral implantation. Predecessors to the INTRAPEC have been employed postoperatively with some success, suggesting proof of concept.^5,6^ However, these techniques have only been investigated for postoperative pain and spasm control. Neither of these techniques have been formally studied for preoperative employment for the purposes of postoperative spam control effectiveness.

## MATERIALS AND METHODS

### Setting and Subjects

Enrollment and data collection took place at the office-based surgical center. This center has 2 operating areas and various patient care and screening regions. The center functions as a cosmetic and wellness office, as well as fully functioning surgical theater. Following study approval, women scheduled to undergo subpectoral implantation under general anesthesia, with airway maintenance via a laryngeal mask airway and combined erector spinae/INTRAPEC regional blockade for intra- and postoperative analgesia were asked to participate. All participants were physically qualified for office-based breast surgery, and thus were physical status 1 or 2 only, with no allergies to medications used in the protocol. Informed consent for anesthesia included verbal understanding of the risks, benefits, and alternatives to general anesthesia with regional blockade including the intrapack technique. Additionally, informed consent for data collection included verbalized understanding of the risks/benefits/alternatives to providing data about pain and spasm for the 48 hours after surgery. Lastly, an explanation regarding the distinction between pain, such as burning or stinging pain associated with the incision, and muscle spasm, eg, the uncontrollable sensation and irritation of the pectoralis muscle contractions, was provided. All study participants demonstrated verbal understanding of their contributions to the data collection, specifically, their accuracy in record production.

### Procedures

After informed consent for surgery, anesthesia, and study participation, patients were escorted to the OR and assisted into a comfortable prone position. Full monitors were placed and 2 mg of IV Midazolam were administered. Each patient received an ultrasound-guided erector spinae plane (ESP) block to match their surgical region of interest. This emphasized a targeted approach, modified from the original work by Forero.^7^ Following the ESP blocks, the patients were assisted into a supine position and preoxygenated. General anesthesia was induced, and a laryngeal mask airway placed. The anterior chest was exposed and every effort made to preserve the surgeons markings. A covered linear probe was paired with a Terason 3300 (Teratec, Burlington, Mass.) and used to obtain a transverse view of the pectoralis major muscle. Attention was paid to the surrounding deeper structures, noting the lung pleura and known regional vasculature. If an existing subpectoral implant were present, it was sonographically mapped to mark its position under the muscle and observe its casual dimensions. The general condition and any notable attributes of the musculature were reported to surgeon. Upon location of the largest and most inferior portion of the pectoralis major muscle, skin prep was applied again to the needle entry point. A 20-gauge block needle (SonoBlock, PAJUNK, Geisingen, Germany) was introduced, in plane, directly into the thick portion of the muscle. Total doses of low concentration local anesthesia varied slightly depending on safe total dosing guidelines, based primarily on weight and remainder of local anesthetic following completion of the ESP block. All patients received at least 15 mL of total volume for the INTRAPEC. The final concentration of each INTRAPEC injection was ropivacaine 0.25% and lidocaine 0.5% with epinephrine 1:200,000 into each muscle belly. Patients also received 4 mg of IV Zofran and 10 mg of IV Decadron as part of a multimodal anesthesia plan. Sequential compression devices were applied to the lower extremities, and the surgeon then proceeded to create a subpectoral dual plane using a lighted retractor and placed the final breast implants. Care was used to avoid injury to the medial pectoral, lateral pectoral nerve. And finally, the subpectoral implant was placed and surgery completed. Spontaneous ventilation was maintained throughout, and no opiates were administered to any patient. Postoperatively, patients were transferred to the postanesthesia care unit and observed until ready for discharge to home. Patients were provided with oxycodone/APAP (5/325 mg tablet, 1 tablet q 6 hours) as needed for analgesia and cyclobenzaprine (10 mg tablet PO q 8 hours) as needed for muscle spasms that occurred despite ESP/INTRAPEC block.

**Fig. 1.**
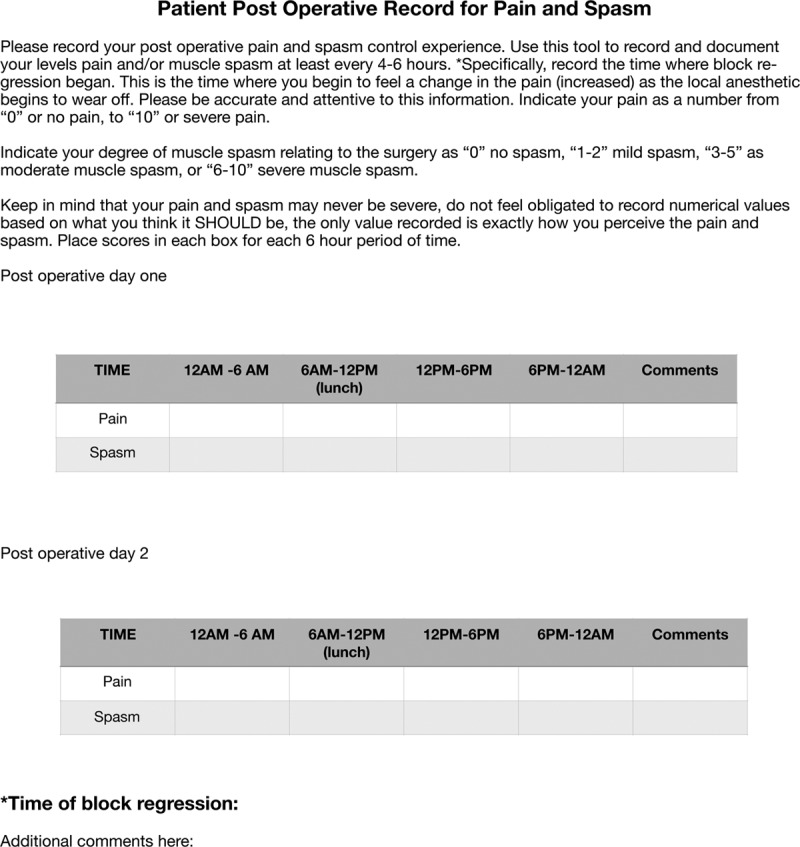
Actual spasm/pain tool example.

After discharge, study participants were asked to differentiate, rate, and record their severity of surgical pain and muscle spasm every 6 hours for 48 hours using 2 separate numeric rating scales on a provided form (Fig. 1). Pain and spasm were each rated on scales from 0 to 10 (0 = “no pain/spasm”; 10 = “as much pain/spasm as you can imagine”). Additional formalized space for open-text comments was also provided, and patients were encouraged to write down notes about their relevant experiences for discussion at the postoperative visit. The pain/spasm tools were then collected upon their first postoperative visit, which occurred on postoperative days 2–4.

### Data Analysis

Data were transcribed from the collection sheets into a Microsoft Excel spreadsheet, without any HIPAA identifiers and presented to a statistics specialist. After transcription, descriptive statistics were generated to characterize the centrality and dispersion of the data, and box plots were generated to depict pain and spasm scores over time.

## RESULTS

**Fig. 2.**
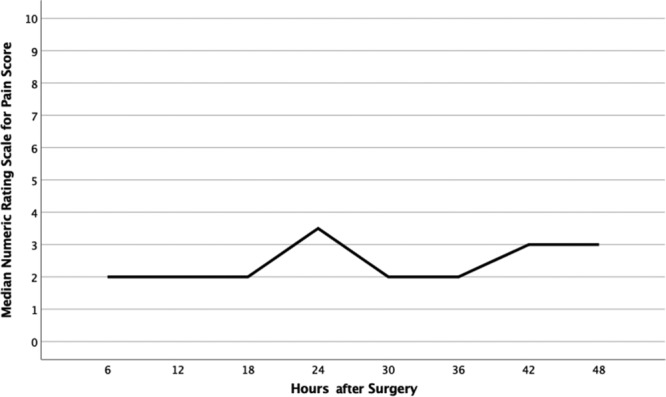
Median Numeric Rating Scale for Pain scores over 48 hours after surgery.

**Fig. 3.**
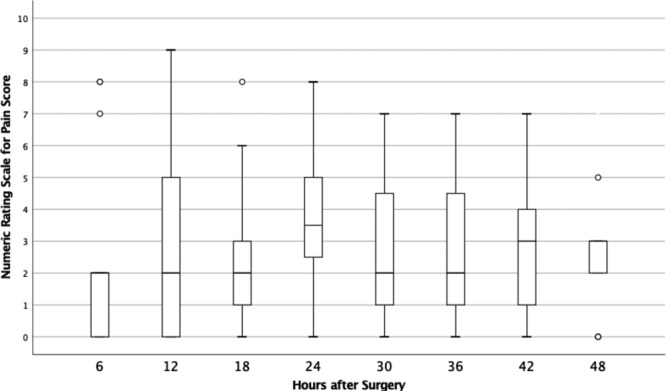
Numeric Rating Scale for Pain scores over 48 hours after surgery. Circles denote outliers.

**Fig. 4.**
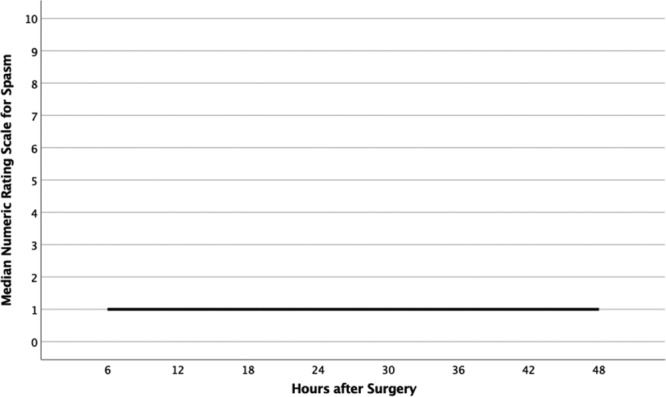
Median Numeric Rating Scale for Spasm scores over 48 hours after surgery.

**Fig. 5.**
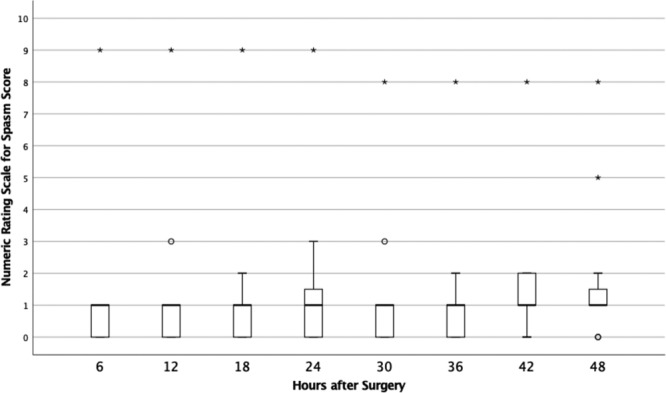
Numeric Rating Scale for Spasm scores over 48 hours after surgery. Circles denote outliers, and stars denote extreme outliers.

A total of 25 women aged 22–70 agreed to participate in data collection. In total, 17 of the 25 patients returned the spasm and pain tool at their first postoperative visit. The 48-hour postoperative scores for pain and spasm are depicted in Figures 2 and 3. At most time points, patients reported a median score for pain of 2, with IQRs ranging from 1 to 7. The highest median pain score was reported at 24 hours after surgery (median, 3.5; IQR, 3). Median spasm score was 1 at every time point after surgery, with IQRs ranging from 1 to 2. Of the 17 study participants, 13 (76.4%) reported spasm scores less than 3 for the entire 2-day study period. Figure 4 shows results of the median numeric rating spasm score, collected over 48 hours immediately after surgery. Figure 5 shows the actual numeric rating scale for spasm over 48 hours after surgery. The circles denote outliers, and stars denote extreme outliers.

## DISCUSSION

One fairly unrecognized advantage of the INTRAPEC injection is muscle compliance during the surgical subpectoral implantation procedure. Following preprocedural INTRAPEC injection, the pectoralis major muscle itself remains pliable and compliant while being surgically manipulated. This is a valued asset in the surgical field during implant placement and sizing.^1^ These desirable elements can be accomplished without the need for parenteral muscle relaxants, negating the need for tracheal intubation in many patients and making opioid-free anesthesia more prolific for patients to experience, and easier for anesthesia providers to employ.

We recognize that there are several faults to the study. Some patients have an inherent difficulty in differentiating pain from spasm despite a thorough explanation. This may account for the outlier data set. The data collection is entirely dependent on patients’ mindful and careful participation. We found that many patients returned incomplete records or failed to complete records altogether. This made the overall study population reduced. The primary study investigator was also responsible for performing all of the ultrasound-guided regional techniques. This could potentially introduce bias. The data collection tool asked patients to record their experiences every 6 hours, which, while reasonable, is not as precise as recording every hour. However, from a practical standpoint, asking patients to record values hourly seemed too cumbersome and would promote inaccuracies. Many implant recipients, despite good anesthesia and analgesia, complain of pressure that may be confused with either spasm or pain. Patients who had experienced extensive surgical times, requiring arm abduction, often complained of shoulder pain. The recording of this and other types of non-surgical pain that happened to coincide with pain gathering data, could also affect statistical outcomes.

We also observed that the INTRAPEC technique seems to be effective in causing muscle compliance at relatively low concentrations of local anesthetic. Despite the association of higher concentration of local anesthetics usually needed to cause motor blockade through targeted nerve block techniques, injecting local anesthetic directly into the muscle itself was responsive to low concentrations.

It is also recognized that spasm relief may also be tied to pain relief.^8^ As the pain scores were not the primary outcome, we did not include their formal investigation in the Results section but made the causal observation that the patients who recorded low spasm scores also tended to record low pain scores over time. While the pain generators for incisional pain are supposedly covered with the ESP block, it is unlikely that the spasm control is affected by the ESP technique. We also recognize that some postmastectomy patients may have denervation caused by surgical damage. These patients may inherently record either higher scores caused by chronic pain or lower scores caused by nerve dysfunction. Additionally, due to its retrospective nature and simple design, the study was not weighted against a control group and would be less statistically relevant as a scientific effort.

Finally, to our knowledge, the INTRAPEC injection for pectoralis major spasm control remains the only technique allowing for effective intraoperative and postoperative isolated relief for this application of subpectoral implantation.

## CONCLUSIONS

The study results support the notion that the ultrasound-guided INTRAPEC injection, placed before the surgery, satisfactorily controls pectoralis muscle spasm for subpectoral implantation for cosmetic or restorative surgeries of the breast. We are hopeful that further studies will examine a more detailed investigation of the technique and find similar positive results.

## ACKNOWLEDGEMENTS

The authors would like to formally recognize Dr. Wayne Lee, MD, for his willingness to permit and support this effort. Special thanks is also extended to Racheal Brodsky, RN, for her assistance and diligence in encouraging patients to follow through with data production and relaying the data to the primary investigator.
